# Pneumatosis Intestinalis and Pneumoperitoneum After Lung Transplantation: Single-Center Experience and Systematic Review

**DOI:** 10.1155/2024/8867932

**Published:** 2024-10-17

**Authors:** Hiroshi Kagawa, Masashi Furukawa, Ernest G. Chan, Matthew Morrell, Pablo G. Sanchez

**Affiliations:** ^1^Department of Cardiothoracic Surgery, University of Pittsburgh Medical Center, Pittsburgh, Pennsylvania, USA; ^2^Division of Cardiothoracic Surgery, Department of Surgery, University of Utah School of Medicine, Salt Lake, Utah, USA; ^3^Division of Pulmonary, Allergy and Critical Care Medicine, Department of Medicine, University of Pittsburgh Medical Center, Pittsburgh, Pennsylvania, USA; ^4^Division of Pulmonary Medicine, Department of Internal Medicine, University of Utah School of Medicine, Salt Lake, Utah, USA

**Keywords:** pneumatosis intestinalis, pneumoperitoneum; lung transplantation

## Abstract

**Background:** Pneumatosis intestinalis (PI) and pneumoperitoneum are some of the complications after lung transplantation (LT). But only limited reports are published. The purpose of this study is to review our experience and perform a systematic review to discuss the possible causes, risk factors, and management.

**Methods:** We reviewed the characteristics, management, and outcome of the patients who developed PI or pneumoperitoneum after LT in our institution from 2013 to 2022. We also performed a systematic review to discuss the management and outcome.

**Results:** PI and pneumoperitoneum were found in 15 out of 729 patients (2.06%) in our institution. We also found 50 patients in the systematic review. Tracheostomy was performed in 40% and gastrointestinal procedures were performed in 55.6%. Laparotomy was performed in 23.4%. A total of 44.6% of patients had benign physical exams or no symptoms. Rejection was seen in 42.9%. A total of 28.6% of patients died during follow-up periods.

**Conclusions:** This report has the largest number of patients so far with PI and pneumoperitoneum after LT. These conditions have a high rejection and high mortality rate. Mechanical ventilation, tracheostomy, gastrointestinal procedure, CMV infection, *Clostridium difficile (C. difficile)* infection, and immunosuppression can be the risk factors, and the management includes laparotomy or conservative management. It is generally recommended to proceed with laparotomy if patients have portal venous gas, elevated white blood cell count, elevated lactic acid level, decreased bicarbonate level, elevated amylase level, metabolic acidosis, abdominal tenderness, or abdominal distension. Otherwise, most of the patients recover with conservative management with nil per os (NPO), metronidazole, ganciclovir, antibiotics, high-flow oxygen, and holding mycophenolate mofetil (MMF).

## 1. Introduction

Pneumatosis intestinalis (PI) is a pathology which is defined as gas accumulation within the bowel wall [1]. It can happen in the submucosa or subserosa of the small or large intestine [2, 3]. PI and pneumoperitoneum are one of the complications seen after lung transplantation (LT) in adult patients. The incidence of PI is reported to be about 2%–5% [1–3]. PI and pneumoperitoneum were rare but critical condition especially after bilateral LT. There is no consensus as to the risk factors or causes, and there are no protocols or guidelines of management of these conditions. Although there are some case reports [4–6] and case series of this condition [1–3], to the best of our knowledge, this study has one of the largest numbers of patients of PI and pneumoperitoneum after LT.

PI is known to be associated with a wide variety of diseases from benign to life-threatening conditions. Hence, the management can vary from conservative management to urgent surgery. PI is most commonly caused by intestinal ischemia, necrotizing enterocolitis, mesenteric vascular disease, and intestinal obstruction [2]. Uncommonly, benign PI is found incidentally on plain radiograph or computed tomography (CT) and has been reported in association with chronic immunosuppression, solid organ transplantation, bone marrow transplantation, graft versus host disease (GVHD), complication of endoscopy, medication-induced pneumatosis, systemic diseases (e.g., lupus, scleroderma, or acquired immunodeficiency syndrome), inflammatory bowel diseases, pulmonary diseases (chronic obstructive pulmonary disease [COPD], asthma, or cystic fibrosis), or infection [2–4, 6].

The purpose of this study is to review our experience of PI and pneumoperitoneum after LT in adult patients and describe the clinical features, management, and outcomes. We also performed a literature review to investigate the clinical features and management of these conditions.

## 2. Patients and Methods

The Institutional Review Board approved this study, and specific informed consent was waived because of the determination of minimal risk to those patients. We used the prospectively collected database which includes all lung transplant patients at the University of Pittsburgh Medical Center. We identified all patients who underwent LT and subsequently developed PI or pneumoperitoneum. Demographics and baseline variables including age, sex, diagnosis, surgical strategy, diet, tracheostomy, percutaneous endoscopic gastrostomy (PEG), abdominal symptoms, surgical findings, distribution of PI, laboratory values, concurrent infections, immunosuppressive regimens, and outcomes were collected from our database and the electronic medical record. The systematic review was also performed utilizing PubMed/MEDLINE and Embase with search terms including “PI,” “pneumoperitoneum,” and “LT.” Search results were reviewed by each author to ensure inclusion of all relevant articles. Studies including pediatric populations were excluded.

## 3. Results

From January 2013 to December 2022, we performed 729 LT at the University of Pittsburgh Medical Center. Among those, 15 patients (2.06%) were identified to have PI or pneumoperitoneum after LT. The clinical outcomes of all study patients were followed to the study end on February 28, 2023. One patient developed PI before LT when he was on veno-venous extracorporeal membrane oxygenation (VV ECMO) preoperatively. His PI resolved before LT. There was no recurrence of PI on this patient after LT. We excluded this patient from the study. The mean age was 49.3 years, ranging from 20 to 68 years. There were 11 males and 4 females. The primary diagnosis for LT was scleroderma (*n* = 3), idiopathic pulmonary fibrosis (IPF) (*n* = 3), interstitial lung disease (ILD) (*n* = 3), bronchiectasis (*n* = 1), cystic fibrosis (CF) (*n* = 1), pulmonary veno-occlusive disease (*n* = 1), pulmonary hypertension (*n* = 1), GVHD (*n* = 1), and bronchopulmonary dysplasia (*n* = 1). Among those 15 patients, 2 patients were on veno-arterial extracorporeal membrane oxygenation (VA ECMO) preoperatively and 1 patient was on VV ECMO preoperatively. VA ECMO was used in 10 patients during transplant surgery, cardiopulmonary bypass was used in 4 patients, and one patient did not require any mechanical support during transplant surgery. One patient who was not on VV or VA ECMO preoperatively was on VA ECMO postoperatively, and patients who were on VA ECMO preoperatively did not require any mechanical support postoperatively. One patient required VV ECMO postoperatively, and this patient was on VV ECMO preoperatively. The onset and resolution of PI or pneumoperitoneum were defined with radiographic findings. The mean duration from transplant surgery to onset of PI was 32.5 days, ranging from 6 to 133 days. Two patients developed colonic ileus before PI. The mean duration of PI from onset to resolution was 5.79 days, ranging from 2 to 19 days. Pneumoperitoneum was seen in 4 patients, and portal venous gas was seen in 1 patient. PI involved the small intestine in 4 patients (26.7%), cecum in 6 patients (40%), ascending colon in 9 patients (60%), transverse colon in 6 patients (40%), and descending colon in 3 patients (20%). Everyone except one case was made nil per os (NPO) when they were diagnosed as PI. Although this one patient developed PI, he was not made NPO as he had benign physical exam. Tracheostomy was placed in 7 patients (46.7%). Among them, 6 had tracheostomy placement after transplant surgery and before developing PI (40%). One patient had tracheostomy placement after developing PI. One patient received PEG placement before transplant surgery (6.67%), 8 patients received PEG placement after transplant surgery and before developing PI (53.3%), and 3 patients received PEG placement after developing PI (20%). Eight patients (53.3%) had no symptoms and had benign physical exams, and 7 patients (46.7%) had tenderness or guarding. Exploratory laparotomy was performed on 8 patients (53.3%); among them, 3 patients had benign physical exams, and 5 patients had tenderness or guarding on physical exams. All of the 3 patients who had benign physical exams had no findings of ischemia, necrosis, or any other abnormality during the exploratory laparotomy. Among the 5 patients who had tenderness or guarding and underwent exploratory laparotomy, 2 patients had no findings during the laparotomy. Among other 3 patients, ileal resection and right hemicolectomy, followed by total colectomy, were performed in one patient due to ischemia; resection of the small intestine and right hemicolectomy were performed in one patient due to ischemia; and another patient had ischemic cecum with perforation and right hemicolectomy with end ileostomy was performed. One of the 2 patients with tenderness on physical exams did not have exploratory laparotomy because of the normal white blood cell (WBC) count and the normal serum lactate level. Although another patient had a normal serum lactate level, the WBC count was high. However, he did not have exploratory laparotomy for unknown reason. His PI resolved in 3 days which was confirmed with CT. *Clostridium difficile* (*C. difficile*) infection was seen only in 3 patients, and cytomegalovirus (CMV) infection was seen in 1 patient. Basiliximab (Simulect) was used as induction in 8 patients, and alemtuzumab (Campath) was used as induction in 7 patients. Everyone was on corticosteroids (prednisone), calcineurin inhibitors (tacrolimus), and antiproliferative agents (mycophenolate mofetil [MMF]) as immunosuppressive therapy after transplant surgery. MMF was held on 13 patients after patients developed PI (86.7%). Eleven patients developed rejection on transbronchial biopsies after transplant surgery (73.3%). No one had recurrent PI during the study period. Six patients died in the follow-up period (40%). Two patients died from multiorgan failure on Postoperative Days (PODs) 9 and 685. One patient died from chronic lung allograft dysfunction (CLAD) on POD 86, 1 patient died from posttransplantation lymphoproliferative disorder (PTLD) on POD 314, 1 patient died from intracranial hemorrhage on POD 334, and 1 patient died from a combination of PTLD and bronchiolitis obliterans syndrome (BOS) on POD 551. The summary of these patients is shown in Table 1.

The systematic review found three case series studies (*n* = 7, 10, and 23) [1–3], and there were 9 case reports [4–12] about PI and pneumoperitoneum after LT. There was a systematic review which included a total of 11 cases of PI after LT [13]. We reviewed all of these studies and reports which had 50 patients in total. Some case reports and the systematic review had overlapped patients. The summary of these patients is shown in Table 2. Not all of the information was available in these 50 patients. Some of them lack the information about tracheostomy, gastrointestinal procedure, duration of PI, distribution of PI on radiographic imaging, laparotomy, *C. difficile* infection, CMV infection, WBC level, lactate level, rejection, or survival. The mean age of the patients was 54.8 years, ranging from 22 to 79 years. There were 31 males and 19 females. The primary diagnosis for LT was IPF (*n* = 24), scleroderma (*n* = 6), emphysema (*n* = 4), CF (*n* = 3), sarcoidosis (*n* = 3), pulmonary GVHD (*n* = 3), COPD (*n* = 2), pulmonary veno-occlusive disease (*n* = 1), pulmonary hypertension (*n* = 1), lymphangioleiomyomatosis (*n* = 1), combined pulmonary fibrosis and emphysema (*n* = 1), and alpha-1 antitrypsin-related COPD (*n* = 1). The mean duration from LT to development of PI was 215.6 days, ranging from 5 to 2495 days. The mean duration from developing to resolution of PI was 34.5 days, ranging from 3 to 69 days. Again, not all of the patients have all of the information in these 50 patients, and some of them have missing data. Pneumoperitoneum was seen in 31 patients out of 49 patients (63.3%), and portal venous gas was seen in 3 patients out of 34 patients (8.82%). PI involved the small intestine in 1 patient (2.1%), cecum in 28 patients (59.6%), ascending colon in 40 patients (85.1%), transverse colon in 29 patients (61.7%), descending colon in 11 patients (23.4%), sigmoid colon in 1 patient (2.1%), and rectum in 1 patient (2.1%). Nothing was mentioned about PI distribution in 3 patients. Management of PI included NPO in 24 patients out of 30 patients (80%), and no information was mentioned in other 20 patients about diet. Seventeen patients out of 30 patients (53.3%) had a PEG, surgical gastrostomy, or jejunostomy tube placement or Nissen fundoplication before developing PI. Again, no information was mentioned about these procedures in other 20 patients. Twenty-two patients (44%) had no abdominal symptoms out of 50 patients. Exploratory laparotomy was performed in 7 patients out of 49 patients (14.2%). One patient has no information whether this patient had laparotomy or not. Among these 7 patients, 1 patient had no symptoms, 1 patient had nausea/vomiting without abdominal pain, and 5 patients had abdominal pain or tenderness. One patient with no symptoms was found to have cecal ischemia and perforation by exploratory laparotomy, and another patient with nausea/vomiting had no abnormal findings during surgery. Among other 5 patients who had abdominal pain or tenderness and underwent exploratory laparotomy, 3 patients had no findings and 2 patients had ischemic colon. *C. difficile* infection was seen only in 1 patient out of 7 patients (14.3%) and CMV infection was seen in 5 out of 41 patients (12.2%). Information about *C. difficile* and CMV was available only in 7 and 41 patients each. Nothing was mentioned about C. *difficile* and CMV in other patients. Four patients had rejection after LT out of 20 patients in which the data on rejection were reported (20%). Outcome of survival was also mentioned only in 20 patients, and 4 patients (20%) died from pulmonary complication, acute myocardial infarction, and unknown reasons.

## 4. Discussion

Our institution identified 15 cases of PI after LT, and we found additional 50 cases from the systematic literature review. We reviewed all of these 65 cases of PI which happened after LT. To the best of our knowledge, this is the largest series of cases of PI after LT.

As mentioned above, PI happens in various conditions and it is known to be associated with a wide variety of diseases from benign to life-threatening conditions. Among these conditions, PI associated with organ transplantation poses a unique challenge since these patients just have undergone an extensive surgery and are on immunosuppression [1]. PI after LT is thought to be associated with steroid therapy, infectious colitis including CMV or *C. difficile*, or GVHD. And there is no guideline about the management of PI on these high-risk patients.

Our data have a similar rate of incidence of PI and pneumoperitoneum to the other reports. However, we believe the true rate of occurrence is much higher. As shown in this report, 29 patients out of 65 patients (44.6%) had benign physical exams or no symptoms. If these patients did not have CT or X-ray studies, we could not identify PI or pneumoperitoneum on these patients. Two patients out of 15 patients in our institution had a colonic ileus that prompted us to get abdominal CT which showed PI. One of them had only bloating and nausea. Even if the patient has only mild symptoms, it is very important to get CT especially after LT not to miss the PI or pneumoperitoneum. Basically, it is very difficult to detect PI with mild symptoms if patients have not imaging studies. We believe we only identify the moderate to severe PI or pneumoperitoneum.

Sometimes, it is not easy to differentiate benign PI from the PI which requires emergent laparotomy from radiological findings. Also, PI after LT is especially challenging to manage as these patients are immunosuppressed and recently underwent transplant surgery. Therefore, these patients may be poor surgical candidates for exploratory laparotomy [2]. However, we suggest presence of abdominal tenderness or abdominal distension, elevated white blood cell (WBC) count, elevated lactic acid level, or presence of portal venous gas warrant an emergent laparotomy. Out of 64 cases (data are missing in one patient about whether she had a laparotomy or not), 15 patients underwent laparotomy (23.4%). Among them, six patients had abdominal tenderness, three patients had abdominal pain, and two patients had abdominal distension. As shown in Tables 1 and 2, seven patients among 15 patients had ischemic changes in the small intestine or colon (46.7%). Only the data from our institution had a WBC level, and the patients who underwent laparotomy had mean WBC of 21.53 (ranging from 5.6 to 53.4) k/*μ*L, and patients who did not undergo laparotomy had mean WBC of 9.3 (ranging from 2.1 to 34) k/*μ*L. This difference was statistically significant. Patients who had ischemic findings on laparotomy had mean WBC of 36.6 (ranging from 15.2 to 53.4) k/*μ*L, and other patients had mean WBC of 10.5 (ranging from 2.1 to 34) k/*μ*L. Although there is no statistical difference on these two groups because of small sample size, patients with ischemic findings had much higher WBC level than another group. Also, the mean lactic acid level of the patients who had laparotomy (1.53 mmol/L) was higher than other patients who did not undergo laparotomy (1.04 mmol/L). Patients who had ischemic findings on laparotomy had a mean lactic acid level of 2.23 (ranging from 1.0 to 4.5) mmol/L, and other patients had a mean lactic acid level of 1.07 (ranging from 0.4 to 1.8) mmol/L. Four patients were found to have portal venous gas among 49 patients (8.16%), and one of four patients (25%) had an ischemic change in her colon which required hemicolectomy. The presence of portal venous gas usually suggests the bowel ischemia [3]. PI with portal venous gas on CT scan is also reported to have an association with bowel ischemia in 70% of patients [6]. These data support the need for a laparotomy. Without abdominal symptoms, elevated WBC count, or elevated lactic acid level, most of the patients can be managed conservatively with supplement oxygen, bowel rest, NPO, and antibiotics [2, 3]. We also recommend to stop MMF as it is known to have gastrointestinal side effect which include nausea, vomiting, diarrhea, abdominal pain, dyspepsia, and anorexia. The reported incidence of gastrointestinal toxicity of MMF is 40%–85% [14]. In our institution, we stopped MMF in 13 patients out of 15 patients on the day when patients were diagnosed to have PI. Two patients were started on azathioprine. Since the discontinuation of MMF can increase the risk of rejection, patients should be monitored closely.

Both of CMV infection and *C. difficile* infection can be the cause of PI. There are some reports mentioning about the relationship between CMV and PI [1, 6, 9, 11, 12]. Inflammation associated with CMV, *C. difficile* or rotavirus infection may cause a break in the integrity of the intestinal mucosa, thus allowing intraluminal air to dissect into the bowel wall [3, 11, 12]. From 56 cases in this study, 6 cases (10.7%) were found to be CMV positive. Three patients died, and one patient had two recurrences of PI. Two patients underwent laparotomy, but there was no specific finding in both cases. Treatment with ganciclovir is necessary if the CMV is positive as CMV infection is associated with significant morbidity and mortality in lung transplant recipients [12]. Early diagnosis, especially for the case of seropositive donor with seronegative recipient, is essential, and it enables us to start virus-specific chemotherapy in time [12]. Infection of *C. difficile* is also reported in PI patients [1, 7]. This study found that four patients were positive for *C. difficile* out of 21 patients (19.0%), two patients died from CLAD, and intracranial hemorrhage. Even though it has low occurrence, we suggest to start metronidazole for the patients with PI even before *C. difficile* test come back due to its high mortality in lung transplant patients.

Also, there may be some associations between immunosuppressive medications, corticosteroids, and development of PI after LT [1, 15]. There is a report that a higher dose of steroids and higher serum levels of tacrolimus are the risk factors for developing PI [13]. We only have the data of prednisone dose and tacrolimus dose from the patients in our institution. And there was no statistical difference of prednisone dose and tacrolimus dose between the patients who had PI and survived and the patients who had PI and died. Further studies are warranted about the relationship between medications, PI, and survival rate.

There are some possible mechanisms of PI in lung transplant patients. These are mechanical, bacterial, biochemical, and immunosuppressive theories [1, 2, 4, 6, 13]. The mechanical theory is that the gas dissects into the bowel wall due to increased intestinal luminal pressure or increased mediastinal pressure via the lungs as a result of mechanical ventilation or emphysema. The bacterial theory is that gas-forming bacteria enter the submucosa through mucosal rents and produce gas within the bowel wall. The biochemical mechanism is that excessive gas produced by bacterial fermentation of carbohydrates in the bowel lumen is absorbed into and trapped inside the bowel wall. Immunosuppressive theory is that immunosuppression-driven atrophy of lymphoid tissues within the bowel wall results in mucosal degeneration and allowing dissection of intraluminal air into the intestinal wall and causes PI [2, 3]. This atrophy may compromise the integrity of the bowel mucosa and allows gas to dissect into the bowel wall [2]. Another possible mechanism is iatrogenic mucosal injury due to feeding tube, PEG, or any gastrointestinal tract instrumentation. Because of immunosuppression, patients' tissue is friable and prone to be damaged easily. Among the 45 patients who we can get data about PEG placement, 25 patients (55.6%) had PEG placement before developing PI after LT. Also, our data showed six patients out of 15 patients (40%) had tracheostomy before developing PI and after LT. PEG placement and tracheostomy may be the risk factors of PI after LT. Or these patients are sicker at baseline and more likely to develop PI. There is a report from the Eastern Association for the Surgery of Trauma (EAST) pneumatosis study group in 2013 which described about the risk factor for PI in general population [16]. These include presence of hypotension or vasopressor use, peritonitis, a lactate level greater than 2 mmol/L, acute renal failure, and active mechanical ventilation [16]. The lung transplant recipient is obviously different from the general population who was in this report; however, duration of mechanical ventilation is usually long and some patients were on ventilator even before LT. Our data showed the mean duration from surgery to extubation was 4.57 days, ranging from 1 to 8 days. However, tracheostomy was performed since we could not extubate and patients were on mechanical ventilation after tracheostomy for longer period of time. So, the procedure such as tracheostomy itself may not be the risk factor but the mechanical ventilation itself may be the risk factor. Extubating as soon as possible after LT may decrease the risk of PI.

The extent of PI varies a lot. Sometimes it involves only the cecum, and sometimes PI happens in the whole colon. Information about the PI distribution was available in 61 patients and not available in four patients. The data showed PI happens most commonly in the ascending colon (80.3%), followed by the transverse colon (57.4%), cecum (55.7%), descending colon (23.0%), and small intestine (8.20%). However, this extent of PI does not necessarily correlate with the severity of the symptoms or underlying disease [3].

Rejection happened in 15 patients among 35 patients (42.9%) in which data on rejection were reported. Also, 10 patients died among 35 patients (28.6%) in which data on mortality were reported. Once patients develop PI after LT, they have a high chance of rejection and high mortality. This may be partially related with the discontinuation of MMF.

If patients have benign physical exams and reassuring laboratory results, they will most likely recover by conservative management only. Even if patients have pneumoperitoneum, most of them do not require surgical exploration. In these cases, free air in the abdomen probably represents the dissection of sterile intramural gas through the serosal surface of the intestine without causing bowel wall perforation [6]. Pneumoperitoneum without concerning physical exams or laboratory results may not warrant surgical management. Lee et al. reported a simple scoring to predict the mortality of patients with PI [17]. According to this report, a bowel wall enhancement pattern on CT and signs of peritoneal irritation were the most important factors to predict the outcome. The decreased or absent contrast enhancement on CT and presence of a peritoneal irritation sign on physical examination associate with a significant increased risk of mortality [17]. However, the PI after LT is unique from these conditions as patients are on immunosuppression, prone to have infection and have little reserve. Also, these patients are generally high risk operative candidates owing to the likelihood of poor wound healing from chronic immunosuppression and are at a higher risk of intraoperative and postoperative complications [10]. Therefore, we cannot simply apply this scoring prediction to the patients with PI after double LT.

We suggest to hold MMF, decrease dose of steroids, and start additional immunosuppression such as azathioprine when we manage the patient conservatively. Also, patients need to be treated with NPO and metronidazole to cover *C. difficile*, gram-negative bacteria, and anaerobes. High-flow oxygen is considered to be effective as well. This approach is based on the theory that inhalation oxygen is forced into the hydrogen containing PI by diffusion and decrease the partial pressure of nonoxygen gases in the venous system, which promote hydrogen diffusing out of the bowel wall and oxygen eventually being reabsorbed [10, 18]. There are several laboratory values that can help predict a prognosis in patients with PI after LT. These include a lactic acid level >2.0 mmol/L, bicarbonate <20 mEq/L, amylase >200 U/L, and pH level <7.3 [10, 19]. Even though post–lung transplant patients are high risk for exploratory laparotomy, we still suggest to proceed with surgery if patients have portal venous gas, elevated WBC count, elevated lactic acid level, or abdominal tenderness or abdominal distension. Some reports mentioned about the factors associated with the need to undergo laparotomy [16, 18]. These were presence of hypotension, acute abdomen on physical examination, elevated lactate level, low serum bicarbonate level, elevated WBC count, age above 60 years, acute rise of creatinine, ascites, portal venous gas, or bowel dilatation on CT [18]. These studies were performed on general population, and we have to be cautious when we apply these parameters as LT recipients have different characteristics from general population. Even though this study has the largest number of PI patients after LT, it is still only 65 cases and not enough cases to make concrete recommendations. Also, even though our experience of PI after LT happened only after bilateral LT at the University of Pittsburgh Medical Center, most of the reported cases did not mention about single LT or bilateral LT. This means there are no data to mention about whether PI depend upon laterality or not. Due to these limitations, further studies are warranted.

## 5. Conclusions

In conclusion, we summarize the PI after LT using our own data and systematic review. To the best of our knowledge, 65 patients is the largest number of PI after LT. Mechanical ventilation, tracheostomy, gastrointestinal procedure, CMV infection, *C. difficile* infection, and immunosuppression can contribute to the development of PI after LT. Although most of the patients recover only with conservative management with NPO, metronidazole, ganciclovir, antibiotics, holding MMF, and high-flow oxygen, there are still some patients who require exploratory laparotomy due to the ischemic small intestine or colon. We recommend laparotomy if patients have portal venous gas, elevated WBC count, elevated lactic acid level, decreased bicarbonate level, elevated amylase level, metabolic acidosis, abdominal tenderness, or abdominal distension. Pneumoperitoneum itself does not warrant a laparotomy. Patients with PI after LT have a high rejection rate (42.9%) and high a mortality rate (28.6%). Most importantly, management of PI after LT needs to be discussed in a multidisciplinary team involving general surgery, infectious disease, transplant pulmonology, and thoracic surgery. Further studies are warranted to discuss the diagnosis and therapeutic management protocol.

## Figures and Tables

**Table 1:** Patient characteristics at our institution.

**Patient**	**Age**	**Sex**	**Diagnosis**	**Pre-Op ECMO**	**Post-Op ECMO**	**Tracheostomy (POD)**	**PEG (POD)**	**PI (POD)**	**Duration of PI**	**PI distribution**	**Pneumo peritonium**	**portal venousgas**	**Duration of NPO**	**Physical Exam**	**Ex-Lap (POD)**	**Ex Lap Findings**	**WBC**	**Lactate**	**C-diff**	**CMV**	**MMF held?**	**Rejection**	**Survival**	**Died on POD**	**Cause of Death**
1	20	M	Bronchopulmonary Dysplasia	No	VA	12	58	19	7	S/A	No	No	No NPO	Benign	No		7.4	1.8	Yes	Yes	Yes	Yes	No	86	CLAD
2	46	M	ILD	No	No	7	26	31	4	A/T	No	No	7	tenderness	31	No findings	14.6	1.1	No	No	Yes	Yes	Yes		
3	60	F	Bronchiectasis	No	No	No	No	6	7	Cecum	No	No	6	tenderness	6, 9	Ileal resection, right hemicolectomy	41.3	1.2	Yes	No	Yes	No	Yes		
4	62	M	Secondary Pulmonary Hypertension	No	No	No	12	25	12	C/A	No	No	11	tenderness	1/26/00	No findings	5.6	1.3		No	Yes	No	Yes		
5	54	M	IPF	No	No	5	30	30	3	A/hepatic flexure	No	No	2	Benign	No		6.5	0.6	No	No	No	Yes	Yes		
6	52	M	GVHD	No	No	5	No	36	17	A/T/D	No	No	18	Benign	No		5.1	0.4	No	No	Yes	Yes	Yes		
7	51	F	Scleroderma	VA	No	No	No	8		S/C	Yes	No	No NPO	distended and tense	8	small intestine resection, right hemicolectomy	53.4	4.5	No	No	No	No	No	9	Multiple organ faliure
8	62	M	IPF	No	No	No	16	12	19	A/T	No	Yes	8	Benign	21	No findings	22.2	1.1	No	No	Yes	Yes	No	314	PTLD
9	35	F	Pulmonary Veno- Occlusive Disease	No	No	16	8	8	4	A	Yes	No	8	tenderness, guarding	8	R colectomy	15.2	1	No	No	Yes	Yes	No	685	Multiple organ failure
10	42	M	Scleroderma	No	No	No	13	30	6	S	No	No	5	Benign, bloating, nausea	No		2.9	0.9	No	No	Yes	Yes	Yes		
11	68	M	ILD	No	No	No	20	13	3	C	No	No	3	tenderness	No		34	1.4	No	No	Yes	Yes	Yes		
12	44	M	Scleroderma	VA	No	No	18	46	2	C/A/T/D	Yes	No	1	Benign	46	No findings	7.9	1.4	No	No	Yes	Yes	Yes		
13	22	M	CF	VV	VV	8	pre-op	61	6	A/T/D	No	No	4	Benign	No		8.9	1.1	No	No	Yes	Yes	No	551	BOS, PTLD, Liver dysfunction, ESRD
14	53	M	ILD	No	No	20	42	133	3	S	No	No	4	tenderness	No		7.8	1.1	Yes	No	Yes	No	No	334	intracranial hemorrhage
15	68	F	Pulmonary fibrosis	No	No	No	13	29	5	C/A/T	Yes	No	5	Benign	29	No findings	12	0.7	No	No	Yes	Yes	Yes		

Abbreviations: A, ascending colon; BOS, bronchiolitis obliterans syndrome; C, cecum; C-dif, *Clostridium difficile* infection; CF, cystic fibrosis; CLAD, chronic lung allograft dysfunction; CMV, cytomegalovirus infection; D, descending colon; ECMO, extracorporeal membrane oxygenation; ESRD, end stage renal disease; Ex-Lap, exploratory laparotomy; F, female; GVHD, graft versus host disease; ILD, interstitial lung disease, IPF: idiopathic pulmonary fibrosis, Lac: lactate level, M: male, MMF: mycophenolate mofetil, NPO: nil per os, PEG: percutaneous endoscopic gastrostomy; PI, pneumatosis intestinalis; POD, postoperative day; PTLD, posttransplantation lymphoproliferative disorder; S, sigmoid colon; T, transverse colon.

**Table 2:** Patient characteristics from the systematic review.

**Patient**	**Age**	**Sex**	**Diagnosis**	**Gastrointestinal Procedure**	**PI (POD)**	**Duration of PI**	**PI distribution**	**Pneumoperitoneum**	**Portal venous gas**	**Duration of NPO**	**Symptom**	**Physical Exam**	**Ex-Lap**	**Ex-Lap Findings**	**Lac**	**C-diff**	**CMV**	**Immunosuppression**	**Rejection**	**Survival**		

1	39	F	Scleroderma	Surgical gastrostomy tube or jejunostomy tube, Nissen fundoplication	84, 137, and 590	16, 25, and 4	C/A/T/D	No		5	Diffuse abd pain, nausea and vomit	Mild tenderness	Yes	Diffuse colonic PI and extensive mesenteric gas	1.3	No	Yes	AZA + P + TA	Yes	No		
2	64	M	Pulmonary fibrosis	Nissen fundoplication	453	5	C/A	Yes		5	Abd pain and nausea	Benign	No		1.1			P + TA	No	Yes		
3	51	M	CF		128	35	C/A/T/D	Yes		5	None	Benign	No		Normal	No		AZA + P + TA	No	Yes		
4	55	M	COPD		24	22	C/A/T	Yes		5	Flank pain and diarrhea	Benign	No		1.7			AZA + P + TA	No	Yes		
5	65	M	Pulmonary fibrosis	Surgical gastorostomy tube or jejunostomy tube, nissen fundoplication	125	13	C/A	No		5	None	Benign	No		1.3			AZA + P + TA	No	No		
6	22	F	CF	Fluoroscopy gastrojejunostomy tube, nissen fundoplication	16	69	C/A	No		5	abd pain	Benign	No		Normal	No		AZA + P + TA	No	Yes		
7	59	M	Pulmonary fibrosis	Surgical gastorostomy tube or jejunostomy tube, nissen fundoplication	83	14		Yes		5	Left shoulder pain radiating abd	Benign	No		0.7			AZA + P + TA	No	Yes		
8	58	M	Sarcoidosis	PEG	23		C/A	Yes	No	2	abd pain and diarrhea		No		23.5		No	MMF + P + TA				
9	43	M	IPF	PEG	47		C/A/T/D	No	No	4	None		No		1.9		No	MMF + P + TA				
10	71	F	IPF	PEG	53		C/A/T	No	No	3	Nausea, vomit, and diarrhea		No		1.4		No	MMF + P + TA				
11	66	F	Sarcoidosis	PEG	36		A/T	Yes	No	1	Nausea and vomit		Yes	No findings	1.6		No	MMF + P + TA + IVIG				
12	67	F	IPF		517		A	Yes	No	0	Diarrhea		No		0.7		No	MMF + P + TA				
13	66	F	IPF		147		C/A	No	No	2	abd pain, nausea, and vomit		No		1.1		No	MMF + P + TA				
14	64	M	IPF		8		C/A	No	Yes	1	Diarrhea		No				No	MMF + P + TA				
15	59	M	Scleroderma and ILD		5		C	Yes	No		None		Yes	Cecal ischemia and perforation			No	MMF + P + TA				
16	69	F	Scleroderma and ILD		41		C/A/T	Yes	No	1	Bloating		No		2.2		No	MMF + P + TA				
17	37	F	Scleroderma and ILD		28		C/A/T/D	Yes	No	1	None		No		0.9		No	MMF + P + TA				
18	79	M	IPF		375		C/A	Yes	No	1	None		No		2		No	MMF + P + TA				
19	54	F	IPF		28		C/A/T/D	Yes	No	0	None		No		1.6		No	MMF + P + TA				
20	71	M	IPF	PEG	209		C	No	Yes	1	Nausea, vomit, and diarrhea		No		1.6		No	MMF + P + TA				
21	61	F	Scleroderma and ILD		47		C/A/T	Yes	No	1	None		No		2.6		No	MMF + P + TA				
22	73	M	CPFE		37		C/A/T	Yes	No	3	Diarrhea		No		3.3		No	MMF + P + TA				
23	61	M	IPF	PEG	63		C/A/T/D	Yes	No	0	Diarrhea		No		1.8		No	MMF + P + TA				
24	59	F	IPF	PEG	234		C/A/T/D	No	No	0	None		No		0.5		No	MMF + P + TA				
25	43	M	IPF	PEG	200		A/T	Yes	No	0	None		No		1.8		No	MMF + P + TA				

**Patients**	**Age**	**Sex**	**Diagnosis**	**PEG**	**PEG before PI**	**PI (POD)**	**Duration of PI**	**PI distribution**	**Pneumoperitoneum**	**Portal venous gas**	**Duration of NPO**	**Symptom**	**Physical Exam**	**Ex-Lap**	**Ex-Lap Findings**	**WBC**	**Lac**	**C-diff**	**CMV**	**Immunosuppression**	**Rejection**	**Survival**

26	71	M	IPF		Yes	24		C	Yes	No	0	None		No			1.5		No	MMF + P + TA		
27	58	M	Alpha-1 antitrypsin-related COPD		No	17		C/A/T/D	Yes	No	1	Diarrhea		No			0.6		No	MMF + P + TA		
28	55	M	IPF		Yes	1477		C/A	No	No	1	None		No			1.5		No	P + TA		
29	69	M	IPF		Yes	51		C/A/T	Yes	No	1	None		No			0.8		No	MMF + P + TA		
30	62	M	IPF		No	202		C/A/T	No	No	1	Emesis		No			1		No	MMF + P + TA		
31	56	M	Emphysema			73	21	A/T/SC	No	No		Vomit and diarrhea	Benign	No		9			No	MMF + P + TA	No	Yes
32	59	M	Emphysema			160	14	C/A/T/D	Yes	No		abd pain	Benign	Yes	Subtotal colectomy and end ileostomy	6.5	4.1		No	MMF + P + TA	No	Yes
33	32	F	GVHD			134	14	A/T	Yes	No		Diarrhea	Benign	No		5.6	1.1		No	AZA + CY + MMF + P	No	Yes
34	59	M	IPF			19	20	A/T	Yes	No		Diarrhea	Benign	No		12	1.2	No	No	MMF + P + TA	No	Yes
35	35	F	Pulmonary veno-occlusive disease			63	15	S	No	No		Diarrhea and abd pain	Rigidity	No		0.4	1.7		No	MMF + P + TA	No	No
36	53	F	Emphysema			5	16	A	No	Yes		Distension	Distension	Yes	Right hemicolectomy and ileostomy	16	10.5		No	MMF + P + TA	No	Yes
37	67.9	M	IPF			90	15	A/T	No	No		None	Benign	No		8.7			No	MMF + P + TA	No	Yes
38	63	F	IPF			455	30	T	Yes	No		None	Benign	No		10.7	1.8		Yes	MMF + P + TA	No	No
39	42	F	Sarcoidosis			2495	17	C/A/T	No	No		None	Benign	No		9.2			No	MMF + P + TA	No	Yes
40	49	M	Emphysema			34	13	A/T	Yes	No		None	Benign	No		8.5			No	MMF + P + TA +		
rATG	No	Yes																				
41	60	M	Pulmonary fibrosis			90,210		T	Yes			abd pain		Yes	No findings	3.5	0.9	No	No	MMF + P + TA		
42	68	M	COPD			49		A	Yes	No		None	Benign	No		Normal	2.6		No	MMF + P + TA		
43	52	F	Scleroderma			60		A	Yes			None		No		Normal	1.6		No	MMF + P + TA		
44	30	M	Cystic fibrosis			10						abd pain, distension, fever, and diarrhea		No				Yes		AZA + CY + P		
45	26	M	Pulmonary GVHD			120		A/T	Yes			None		No						MMF + P + TA		
46	35	F	Pulmonary GVHD			60	8	A/T/D/rectum				Diarrhea						No	No	MMF + P + TA	Yes	
47	59	M	IPF			1031	11	A/T/D	Yes			None		No					Yes	P + TA		Yes
48	54	M	IPF	GJ tube		240	3	A	No			None		No		11.2	Normal					
49	34	F	Lymphangioleiomyomatosis			480	14		Yes			abd pain		Yes	No findings				Yes		Yes	Yes
50	36	M	PHTN			41			Yes			Diarrhea		No					Yes	AZA + CY + P	Yes	Yes

Abbreviations: A, ascending colon; abd, abdominal; AZA, azathioprine; C, cecum; C-dif, Clostridium difficile infection; CF, cystic fibrosis; CMV, cytomegalovirus infection; COPD, chronic obstructive pulmonary disease; CPFE, combined pulmonary fibrosis and emphysema; CY, cyclosporine; D, descending colon; ECMO, extracorporeal membrane oxygenation; Ex-lap, exploratory laparotomy; F, female; GJ, gastro-jejunum; ILD, interstitial lung disease; IPF, idiopathic pulmonary fibrosis; Lac, lactate level; M, male; MMF, mycophenolate mofetil; NPO, nil per os; P, prednisone; PEG, percutaneous endoscopic gastrostomy; PI, pneumatosis intestinalis; POD, postoperative day; rATG, rabbit antithymocyte globulin; T, transverse colon; TA, tacrolimus.

## Data Availability

All of the data are available in the Tables 1 and 2.

## Nomenclature

BOS: Bronchiolitis obliterans syndrome
*C. difficile*: *Clostridium difficile*
CF: Cystic fibrosis
CLAD: Chronic lung allograft dysfunction
CMV: Cytomegalovirus
COPD: Chronic obstructive pulmonary disease
CT: Computed tomography
EAST: Eastern Association for the Surgery of Trauma
GVHD: Graft versus host disease
ILD: Interstitial lung disease
IPF: Idiopathic pulmonary fibrosis
LT: Lung transplantation
MMF: Mycophenolate mofetil
NPO: Nil per os
PEG: Percutaneous endoscopic gastrostomy
PI: Pneumatosis intestinalis
POD: Postoperative day
PTLD: Post-transplant lymphoproliferative disorder
VA ECMO: Veno-arterial extracorporeal membrane oxygenation
VV ECMO: Veno-venous extracorporeal membrane oxygenation
WBC: White blood cell.
